# Breast imaging surveillance after curative treatment for primary non-metastasised breast cancer in non-high-risk women: a systematic review

**DOI:** 10.1007/s13244-018-0667-5

**Published:** 2018-11-08

**Authors:** Jeroen Swinnen, Machteld Keupers, Julie Soens, Matthias Lavens, Sandra Postema, Chantal Van Ongeval

**Affiliations:** 1 Department of Radiology, UZ Leuven, Herestraat 49, Leuven, Belgium; 2 Department of Imaging and Pathology, KU Leuven, Herestraat 49, Leuven, Belgium

**Keywords:** Breast neoplasms, Recurrence, Aftercare, Review, Practice guidelines as topic

## Abstract

**Objectives:**

The article summarises the available guidelines on breast imaging surveillance after curative treatment for locoregional breast cancer.

**Methods:**

A systematic review of practice guidelines published from 1 January 2007 to 1 January 2017 was performed according to PRISMA methodology. The search was conducted for the EMBASE, MEDLINE, Cochrane and Centre for Reviews and Dissemination databases. On 8 July 2018, all included guidelines were updated to the most recent version.

**Results:**

Twenty-one guidelines originating from 18 publishing bodies matched criteria. Publishing bodies consisted of seven governmental institutions, nine medical societies and two mixed collaborations. Publishing boards consisted of six radiological, four oncological, and 11 multidisciplinary teams. Annual bilateral mammography surveillance after breast-conserving therapy was recommended by 17/18 (94.4%) publishing bodies. Annual contralateral mammography surveillance after mastectomy was recommended by 13/18 (72.2%) publishing bodies. Routine use of digital breast tomosynthesis was recommended by 1/18 (5.6%) publishing bodies. Routine breast ultrasound surveillance was recommended by 2/18 (11.1%), deemed optional by 4/18 (22.2%) and not supported by 8/18 (44.4%) publishing bodies. Routine breast magnetic resonance imaging (MRI) surveillance was not recommended by 16/18 (88.9%) publishing bodies, although 6/18 (33.3%) specified subgroups for systematic MRI surveillance.

**Conclusions:**

Annual mammography is currently the ‘gold standard’ for breast imaging surveillance. The role of digital breast tomosynthesis (DBT) remains to be further investigated. Most guidelines do not recommend routine breast ultrasound or MRI surveillance, unless indicated by additional risk factors.

## Introduction

Breast cancer is the most common malignancy affecting European women. In 2012, 458,337 European women were diagnosed with breast cancer [1]. In Belgium, 88.4% of breast cancers are detected at a locoregional disease stage, allowing curative intention to treat in most patients [2]. As a result, a large survivor population has accumulated over time. In the USA alone, it is estimated there were 3,560,570 female breast cancer survivors as of 1 January 2016, a number expected to rise [3]. As recurrence surveillance poses an increasing workload on imaging centres, use of cost-effective follow-up regimens is essential.

In the absence of strong familial or personal risk factors, breast cancer survivors are considered an intermediate risk subgroup for breast cancer recurrence [4]. The term ‘intermediate risk’ is not well-defined among guidelines, ranging between 15 and 30% risk for recurrence [5–7].

Recurrence can present as a true ipsilateral breast cancer recurrence, a new primary breast cancer in the treated breast, a contralateral breast cancer, an axillary recurrence or distant metastases. Yearly locoregional recurrence (LRR) risk is considered 1.0–1.5%, for at least 15–20 years [4].

## Materials and methods

### Search strategy

Screening and selection were conducted according to the guidelines for Preferred Reporting Items for Systematic Reviews and Meta-Analyses (PRISMA) [8]. Only the first author searched the MEDLINE, EMBASE, Cochrane Database of Systematic Reviews (CDSR) and Centre for Reviews and Dissemination (CRD) databases for guidelines published from 1 January 2007 to 1 January 2017. The MEDLINE and EMBASE search was performed through the EMBASE search engine and consisted of the following search string: ‘breast cancer’/exp. AND (‘disease management’/exp. OR ‘tumour recurrence’/exp. OR ‘evaluation and follow up’/exp) AND ‘imaging and display’/exp. All search terms were expanded. Review of the CDSR and CRD databases was performed for the MeSH term: ‘breast neoplasms’. The reference sections from all full-text assessed papers were also manually searched.

### Selection criteria

Inclusion criteria:

- Practice guideline from a medical society or institutional/governmental body.
- Imaging surveillance after curative treatment for primary non-metastasised breast cancer.
- Journal article, web page, abstract, book section.

Exclusion criteria:

- Male breast cancer.
- High risk for recurrence (≥ 20%), including genetic/familial susceptibility.
- Young breast cancer patients, less than 40 years of age at diagnosis.
- Personal history of B3 lesions (i.e. lobular neoplasia, atypical ductal hyperplasia [ADH], flat epithelial atypia [FEA], papillary lesions, etc.).
- History of chest irradiation.

### Data extraction

Data on the following outcomes was extracted only by the first author: publisher; country and nature of the publishing body; guideline target group; month/year published; timing of imaging surveillance onset; frequency of imaging surveillance; timing of screening alteration; recommended imaging continuation after alteration; termination of imaging follow-up; use of mammography after breast-conserving therapy (BCT); use of mammography after mastectomy; use of breast/axillary ultrasound; use of contrast enhanced-magnetic resonance imaging (CE-MRI) and use of other imaging modalities.

## Results

### PRISMA flow diagram

**Fig. 1:**
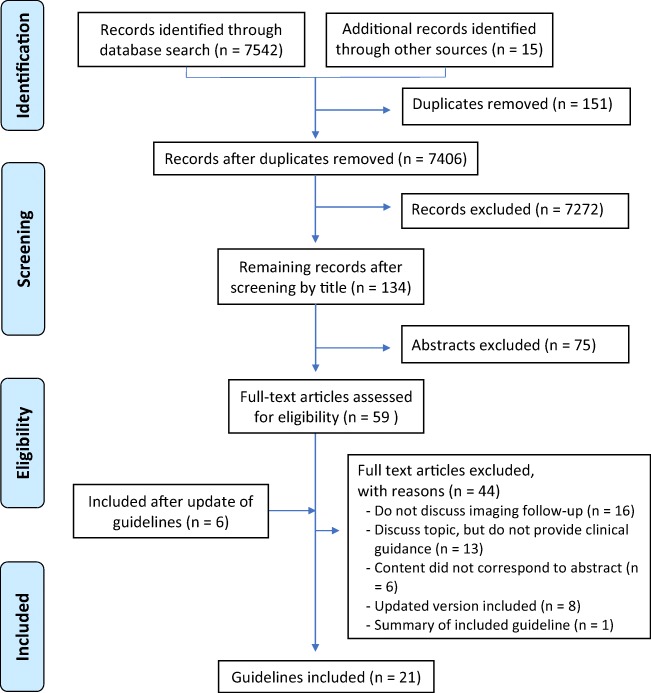
Preferred Reporting Items for Systematic Reviews and Meta-Analyses (PRISMA) flow diagram of systematic search

A PRISMA flow diagram depicts the first author’s article search and selection process (Fig. 1). Out of 7,457 search results, including 151 duplicates, 134 abstracts were evaluated. After full text review of 59 articles and inclusion of 6 updated guidelines, 21 met the inclusion criteria. Of 44 articles rejected, 16 articles did not discuss imaging surveillance after breast cancer, 13 articles discussed imaging follow-up without providing a consensus-based clinical guideline and one article was identified as a summary of an included guideline. For six articles, the content did not correspond to the title. Furthermore, eight articles were excluded because a more recent guideline from the same publishing body was included.

### Characteristics of studies

The 21 included practice guidelines, as described in Table 1, were provided by 18 publishing bodies from the following countries: four from the United States of America (USA), four from Canada, two from the United Kingdom (UK) and one from France, Australia, New Zealand, Germany, Italy, Switzerland, Belgium, and The Netherlands [4, 6, 9–27]. As three guidelines were published by the American College of Radiology (ACR) and two by the Haute Autorité de Santé (HAS), complementary data from these guidelines were combined in Tables 2 and 3, with no conflicting recommendations encountered [6, 9, 10, 19, 20]. Publishing bodies were seven governmental institutions, nine medical societies and two bodies of mixed nature. The British Columbia Ministry of Health-British Columbia Medical Association (BCMH-BCMA) and Nationaal Borstkanker Overleg Nederland-Knowledge Institute of Medical Specialists (NABON-KIMS) guidelines were provided by a governmental body, but received endorsement from their respective medical societies before publishing [14, 22]. Recommendations were made on six guidelines by a radiological, four by an oncological, and 11 by a multidisciplinary board (Table 1). On 8 July 2018, all included guidelines were updated to the most recent version.

**Table 1:** Demographics from included guidelines

Guideline	Reference	Country	Language	Body	Target group	Date
ACR	[6, 9, 10]	USA	English	Medical Society, Radiology	BC [6], Stage I BC [9], BC in women at higher than average risk [10]	Nov 2017 [6], May 2017 [9], Mar 2018 [10]
ACS-ASCO	[11]	USA	English	Medical Society, Oncology	BC	Dec 2015
ASCO	[12]	USA	English	Medical Society, Oncology	Primary BC after curative treatment	Mar 2013
AHS	[13]	Canada	English	Governmental, Multidisciplinary	EBC after BCT	Oct 2015
BCMH-BCMA	[14]	Canada	English	Governmental/Medical Society, Multidisciplinary	DCIS/IBC ≥ 19 years old	Oct 2013
CAR	[15]	Canada	English	Medical Society, Radiology	BC	Oct 2012
CCMB	[16]	Canada	English	Governmental, Multidisciplinary	BC	Jan 2017
DKG-DGGG	[17]	Germany	German	Medical Society, Multidisciplinary	BC	Dec 2017
ESMO	[18]	Switzerland	English	Medical Society, Oncology	Primary BC	Sep 2015
GISMa-ICBR/SIRM	[4]	Italy	English	Medical Society, Radiology	Women with a previous history of breast cancer	Aug 2016
HAS	[19, 20]	France	French	Governmental, Multidisciplinary	BC; BC after curative treatment	Jan 2010 [19], Feb 2015 [20]
KCE	[21]	Belgium	English	Governmental, Multidisciplinary	DCIS / Early IBC	Jul 2013
NABON-KIMS	[22]	Netherlands	English	Governmental/Medical Society, Multidisciplinary	BC (without BRCA 1/2)	Feb 2012
NBOCC	[23]	Australia	English	Governmental, Multidisciplinary	EBC after BCT	Mar 2010
NCCN	[24]	USA	English	Medical Society, Oncology	IBC	Mar 2018
NICE	[25]	UK	English	Governmental, Multidisciplinary	DCIS / Early IBC	Jul 2018
NZGG	[26]	New Zealand	English	Governmental, Multidisciplinary	EBC	Aug 2009
RCR	[27]	UK	English	Medical Society, Radiology	BC (without BRCA 1/2)	Jun 2013

*ACR* American College of Radiology, *ACS* American Cancer Society, *ASCO* American Society of Clinical Oncology, *AHS* Alberta Health Services, *BCMH* British Columbia Ministry of Health, *BCMA* British Columbia Medical Association, *CAR* Canadian Association of Radiologists, *CCMB* CancerCare Manitoba, *DKG* Deutsche Krebsgesellschaft, *DGGG* Deutsche Gesellschaft für Gynäkologie und Geburtshilfe, *ESMO* European Society for Medical Oncology, *GISMa* Italian Group for Mammography Screening, *ICBR* Italian College of Breast Radiologists, *SIRM* Italian Society of Medical Radiology, *HAS* Haute Autorité de Santé, *KCE* Belgian Health Care Knowledge Centre, *NABON* Nationaal Borstkanker Overleg Nederland, *KIMS* Knowledge Institute of Medical Specialists, *NBOCC* National Breast and Ovarian Cancer Centre, *NCCN* National Comprehensive Cancer Network, *NICE* National Institute for Health and Care Excellence, *NZGG* New Zealand Guidelines Group, *RCR* Royal College of Radiologists, *BC* breast cancer, *BCT* breast-conserving therapy, *RT* radiation therapy, *EBC* early breast cancer, *DCIS* ductal carcinoma in situ, *IBC* invasive breast cancer

**Table 2:** Recommendations on onset, frequency, intermediate frequency alteration and termination of breast imaging surveillance

Guideline	Imaging onset	Frequency	Alteration of annual screening frequency	Screening frequency after alteration	Termination of imaging follow-up
ACR	6–12 months after RT	Annual	May be returned to routine screening at some point, dependent upon institutional protocol	Return to routine breast cancer screening	NS
ACS-ASCO	NS	Annual	NS	NS	NS
ASCO	≥ 6 months after RT	Every 6–12 months. Annual if stable mammographic findings	NS	NS	NS
AHS	12 months after diagnosis or ≥ 6 months after RT	Annual	NS	NS	NS
BCMH-BCMA	≥ 6 months after RT	Annual	NS	NS	NS
CAR	NS	Annual	NS	NS	NS
CCMB	12 months after diagnosis or ≥ 6 months after RT	Annual^a^	NS	NS	May be omitted, if life expectancy < 5 years
DKG-DGGG	Dependent on type of RT and/or surgery	Annual	NS	NS	NS
ESMO	NS	Annual	NS	NS	NS
GISMa-ICBR/SIRM	12 months after treatment	NS, but mentions both annual and biannual	NS	NS	Consider stop if > 74 years old and at least 10 years’ follow-up
HAS	≥ 12 months after diagnosis or ≥ 6 months after RT	Annual	NS	NS	Re-evaluate every 5 years
KCE	NS	Annual	Annual at least 10 years	NS	NS
NABON	± 12 months after the last pre-operative mammography/MRI	Annual	After 5 years, if ≥ 60 years old at time of follow-up	Mammography every 2 years^b^	Consider stop if > 75 years old^b^
NBOCC	12 months after diagnosis	Annual	NS	NS	NS^c^
NCCN	6–12 months after RT	Annual	NS	NS	NS
NICE	NS	Annual	After 5 years, if ≥ NHSBSP/BTWSP screening age	NS	NS
NZGG	12 months after diagnosis or 6 months after RT	Every 6–12 months. Annual if stable mammographic findings	NS	NS	NS
RCR	NS	Annual	Reconsider if 50 years old	CL: mammography every 2–3 years IL: mammography every 1–3 years	CL: 75 years old IL: if co-morbidities make detection unhelpful

*NS* not specified, *NHSBSP/BTWS* National Health Service Breast Screening Program/Breast Test Wales Screening Programme. For other abbreviations *see* Table 1

^a^More frequently if recommended by the radiologist

^b^After mastectomy, coordinated by the national breast screening programme. After breast-conserving therapy, coordinated by general practitioner

^c^The duration of the follow-up should be determined in consultation between the physician and patient

**Table 3:** Recommendations on modalities for breast imaging surveillance

Guideline	Mammography		Ultrasound	(CE-)MRI	Other
	BCT	Mastectomy			
ACR	BL^a^	CL	Optional, especially for dense breasts	Recommended for - dense breast tissue - patients diagnosed < 50 years old	DBT^a^
ACS-ASCO	BL	CL	NR	NR	NR
ASCO	BL	NS	NR	NR	NR
AHS	BL	CL	NR	NR	NR
BCMH-BCMA	BL	CL	NR	NR	NR
CAR	BL	NS	NS	NS	NS
CCMB	BL	CL	NR	NR	NR
DKG-DGGG	BL	CL	If quality-assured, should be added for breasts and axilla	May play an additional role in the differentiation of scar vs recurrence	NR
ESMO	BL	CL	BL/CL	May be indicated for young patients, especially in cases of dense breast tissue and genetic/familial predispositions	NR^b^
GISMa-ICBR/SIRM	BL	NS	NS	NR	Brief mention of DBT as a supplemental investigation, without further elaboration or recommendation
HAS	BL	CL	May be associated	NR	NR^c^
KCE	BL	NS	With or without	- Initial BC not seen on other imaging - Other imaging inconclusive	NR
NABON-KIMS	BL	CL	NS	May play an additional role in: - differentiation scar vs recurrence - BC not visible on mammography - autologous breast reconstructions	NR
NBOCC	BL	CL	If indicated on clinical or radiological grounds, including: - young women - dense breasts - initial breast cancer undetectable by mammography	Specific high-risk subgroups	NR
NCCN	BL	CL	NR	NR	NR^d^
NICE	BL	CL	NR	NR	NR
NZGG	BL	NS	NS	NS	NR
RCR	BL	CL^e^	NR	NR	NS

*NS* not specified, *NR* not recommended, *RT* radiation therapy, *LRT* locoregional therapy, *BC* breast cancer, *BCT* breast-conserving therapy, *DBT* digital breast tomosynthesis, *IL* ipsilateral, *CL* contralateral, *BL* bilateral, *(CE-)MRI* (contrast-enhanced) magnetic resonance imaging. For other abbreviations *see* Table 1

^a^Diagnostic digital breast tomosynthesis (DBT) received identical appropriateness score (9/9) and relative radiation level rating (2/3) as diagnostic mammography. For intermediate-risk women, breast mammography or DBT (with accompanying planar or synthesised 2-D images) is recommended

^b^For patients who take tamoxifen, an annual gynaecological examination is recommended, possibly with a gynaecological ultrasound

For patients who take an aromatase inhibitor, regular bone density evaluation is recommended

^c^Depending on the context, following examinations may be indicated: - for patients who take tamoxifen, an annual pelvic ultrasound for excluding endometrial malignancies; - for patients who take an aromatase inhibitor, bone density evaluation every 1–3 years

^d^Monitoring of bone health with a bone mineral density determination at baseline and periodically thereafter is advised for women on aromatase inhibitors or women who experience ovarian failure secondary to treatment

^e^Also ipsilateral, if autologous reconstruction with high recurrence risk

### Imaging onset, frequency and termination

If a patient did not receive adjuvant radiation therapy, 5/18 (27.8%) publishing bodies recommended imaging onset at 12 months after diagnosis. The NABON and GISMa-ICBR/SIRM guidelines recommended onset 12 months after the last pre-operative imaging and after treatment termination respectively. Specification of imaging onset was not provided by 10/18 (55.6%) publishing bodies.

If a patient received adjuvant radiation therapy, 6/18 (33.3%) publishing bodies recommended onset of breast imaging starting at 6 months after completion of radiation therapy. The NCCN and ACR guidelines recommended onset of imaging between 6–12 months post-radiation. The DKG-DGGG guideline stated that onset should be adjusted to the type of surgery and/or radiation therapy, without further specifying a time frame. Specification of post-radiation imaging onset was not provided by 9/18 (50.0%) publishing bodies.

Annual breast imaging surveillance was recommended by 17/18 (94.4%) publishing bodies. In addition, 2/18 (11.1%) guidelines recommended more frequent early follow-up, if postoperative changes impeded recurrence detection. The GISMa-ICBR/SIRM guideline did not provide a recommended screening frequency, but briefly mentioned both annual and biannual imaging follow-up.

Alteration of annual imaging frequency after a certain patient age and/or imaging period, was not discussed by 13/18 (72.2%) publishing bodies. The NICE and NABON guidelines recommended annual imaging for women younger than 50 and 60 years of age respectively. For older women, annual mammography for 5 consecutive years was recommended, before returning to the national breast screening frequency. The Royal College of Radiologists (RCR) recommended annual imaging continuation until 50 years of age, with frequency afterwards varying between 1 and 3 years for ipsilateral and 2–3 years for contralateral surveillance. The Belgian Health Care Knowlegde Center (KCE) stated annual imaging should continue at least 10 years. The ACR recognised patients could return to routine screening at some point, dependent upon institutional protocol.

Termination of breast imaging surveillance was not discussed by 13/18 (72.2%) publishing bodies. The GISMa-ICBR/SIRM and NABON guidelines stated imaging termination should be considered after 74 and 75 years of age respectively. The RCR stated explicitly, no evidence-based recommendation on the timing of surveillance termination could be made at publication. However, the RCR did not recommend routine imaging surveillance of the contralateral breast after the age of 75 years, while routine ipsilateral surveillance should be stopped depending on co-morbidities. The HAS guideline stated imaging surveillance should be re-evaluated every 5 years. The CCMB discouraged imaging screening if life expectancy was less than 5 years.

### Imaging modalities

Following breast-conservative surgery, bilateral mammography was advised by all 18/18 (100%) publishing bodies. If the patient received mastectomy surgery, 13/18 (72.2%) recommended only contralateral mammography follow-up. One exception was made by the RCR, for autologous reconstructions with a high risk for recurrence. Follow-up after mastectomy was not specified by 5/18 (27.8%) publishing bodies.

Routine ultrasound surveillance was not recommended by 8/18 (44.4%) publishing bodies. Furthermore, 4/18 (22.2%) publishing bodies did not recommend routine use, but recognised breast ultrasound as an appropriate screening tool for patients with additional risk factors, such as young age or dense breasts. The only 2/18 (11.1%) publishing bodies that recommended routine use of breast ultrasound, were ESMO and DKG-DGGG, the latter specifically including the axilla. Breast ultrasound surveillance was not discussed by 4/18 (22.2%) publishing bodies.

Routine breast MRI surveillance, with or without intravenous contrast, was not recommended by 10/18 (55.6%) publishing bodies. In the presence of inconclusive findings or additional individual risk factors, 6/18 (27.8%) publishing bodies recognised breast MRI surveillance as a supplemental imaging tool. The ACR recommended systematic use of annual contrast-enhanced MRI in two subgroups of female breast cancer survivors: women with dense breasts and women diagnosed before the age of 50. For these women, MRI should not replace mammography or DBT, but should be used as an adjunct examination. Breast MRI surveillance was not specified by 2/18 (11.1%) publishing bodies.

Only the ACR recognised DBT as a surveillance tool for breast cancer survivors [24]. The ACR considered DBT, with accompanying planar or synthesised 2-D imaging, an equal modality to diagnostic digital mammography. A brief mention of DBT as supplemental imaging modality was provided by the GISMa-ICBR/SIRM guideline, however without further elaboration or recommendation. Use of DBT or other breast imaging modalities was not recommended by 14/18 (83.3%) publishing bodies and not discussed by 2/18 (11.1%) publishing bodies.

## Discussion

### Onset of imaging surveillance

During the first 6–12 months after surgical excision and/or adjuvant radiation therapy (RT), post-surgical and post-radiation changes are most likely to occur [28]. Initial radiological differentiation between scar formation, altered tissue and true recurrence can therefore be challenging. Furthermore, mammography yield during the first months after surgery/radiation therapy appears to be low [29, 30]. Seven publishing bodies therefore recommended a 12-month imaging delay following diagnosis, final pre-operative imaging or treatment. Likewise, six publishing bodies recommended a 6-month and two bodies a 6–12 month imaging delay after radiation therapy (Table 2).

### Screening frequency

Annual breast imaging surveillance was recommended by all included publishing bodies, except for the GISMa-ICBR/SIRM guideline, which did not specify annual or biannual imaging screening. In a recent health technology assessment (HTA), a 12–24 months imaging interval appeared to be most beneficial overall, although women with the lowest recurrence of risk seemed to have the greatest net benefit from a triennial interval. This study also implied that recurrence risk stratification should be considered, to determine the optimal imaging interval [31]. To our knowledge, no prospective study comparing annual to alternative screening frequencies is currently available [4]. A large multi-centre randomised controlled trial (RCT) is currently enrolling, which will compare annual to biennial mammography after BCT and annual to triennial mammography after mastectomy [32].

Although debated, early semi-annual breast surveillance has been found beneficial over annual follow-up [33]. Stabilisation of postoperative mammographic findings, generally takes place 2–3 years after BCT [28]. Two publishing bodies therefore recommended an early 6–12 months imaging interval, if early postoperative changes interfered with recurrence detection.

### Early alteration of annual screening

Optimal duration and frequency of imaging surveillance is a common concern among breast cancer survivors. In a survey by de Bock et al. [34], 56 out of 84 breast cancer survivors responded that they would like to attend lifelong follow-up. Due to paucity of evidence, there is currently no consensus among guidelines for if or when annual imaging should be terminated.

The NICE and NABON guidelines limited the annual screening interval to the first 5 years post-diagnosis, as the rate of true ipsilateral recurrences should peak during this time window [22, 25]. Some authors, however, argue that the combined recurrence rate of true recurrences and new ipsilateral/contralateral breast malignancies, remains steady for at least 20 years [4, 35].

### Termination of imaging surveillance

As the RCR guideline stated, no evidence-based recommendation on the timing of surveillance termination can currently be made [27]. The limited number of recommendations identified, therefore vary widely (Table 2).

### Mammography after breast-conserving therapy (BCT)

All included practice guidelines recommended bilateral mammography surveillance after BCT. Nonetheless, supporting evidence is limited to observational and retrospective findings. Sensitivity ranges between 63.5 and 67%, which is significantly lower than for matched screenings without a personal history of breast cancer, ranging between 73.5 and 76.5% [15–17]. There is no significant difference between ipsilateral and contralateral sensitivity, while specificity ranges 98.2–98.4% [15]. A survival benefit from surveillance mammography in asymptomatic breast cancer survivors has been suggested by multiple observational studies [36]. However, estimated hazard ratio for asymptomatic relative to symptomatic/clinical detection varies widely, ranging between 0.10 and 0.86 [36]. In a single-centre retrospective analysis of 1,044 patients, detection of asymptomatic recurrence improved relative survival between 27 and 47% [37].

### Mammography after mastectomy

None of the included guidelines recommended routine ipsilateral imaging follow-up after mastectomy surgery, with or without reconstruction. This included both implant as well as autologous reconstructive procedures. The RCR was the only body to make an additional recommendation for ipsilateral surveillance in case of an autologous reconstruction with a high risk for recurrence (Table 3). Following a non-reconstructive mastectomy, recurrence should theoretically be limited to the (sub)cutaneous tissue, allowing simple detection from rigorous clinical examination [38]. For implant-based reconstructions, silicone pockets are generally placed behind the pectoralis major muscle, displacing the entire mastectomy site anteriorly. Recurrence should therefore present as superficial and be easily appreciable on clinical examination. In case of an autologous reconstruction, the reconstructed breast comes on top of the major pectoral muscle and is covered by transplanted skin. The true resection margin therefore remains deep to the reconstructed breast [38]. Nonetheless, Lee et al. [39] concluded from a retrospective cohort of 554 mammograms in 265 women who underwent TRAM flap reconstruction that routine mammographic surveillance of all autologous reconstructed breasts was not likely to be beneficial.

### Digital breast tomosynthesis (DBT)

Supplementary or equivalent use of DBT in the general population screening has received much attention in recent literature. Evidence has grown in recent years that DBT addition increases cancer detection and reduces false-positive, recall and interval cancer rates, compared to mammography alone [10]. The ACR therefore recommended DBT with accompanying planar or synthesised 2-D images as a screening tool for breast cancer survivors [6]. However, very few studies have investigated the role of DBT in breast cancer recurrence surveillance [40]. In a prospective single-centre study including 618 women with a personal history of breast cancer, addition of DBT significantly reduced the rate of indeterminate findings from 13.1 to 10.5% (*p* = 0.018) [41]. Nonetheless, concerns remain regarding longer interpretation times, additional dose and effectiveness of 2-D reconstructions [40]. As new evidence emerges, guidelines are likely to undergo revision.

### Ultrasound

As routine ancillary ultrasound surveillance remains debated, most publishing bodies do not recommend routine ultrasound surveillance (Table 3). In the prospective multicentre ACRIN 6666 trial, which included a subgroup of 1,426 female breast cancer survivors with heterogeneously dense breast tissue in at least one quadrant, women were randomised to a sequence of three yearly screenings with mammography alone or a combination of mammography and ultrasound [42]. Subgroup analysis of women with a personal history of breast cancer was included in the supplementary online content. Addition of ultrasound significantly (*p* < 0.001) increased cancer detection from 8.2/1,000 to 12.5/1,000 screens and sensitivity from 55.9 to 84.7%. However, specificity and PPV3 were significantly (*p* < 0.001) inferior after the addition of ultrasound [42].

### MRI

Breast MRI screening, without or with intravenous contrast, is generally limited to high-risk women. Estimated risk to include women in a high-risk screening programme varies widely among guidelines, ranging between 20 and 30% [5–7, 43]. Most women with a personal history of breast cancer, but without explicit familial/genetic/iatrogenic risk of recurrence, are currently considered an intermediate risk subgroup [10]. Breast MRI screening has shown to be cost-effective for high-risk women, but is still debated for intermediate-risk women, although there is growing literature considering this topic.

Additional cancer yield for MRI recurrence surveillance varies between 9.9 to 28.8 cancers/1,000 examinations, but has been reaffirmed in multiple retrospective single-centre studies [40, 44]. In 2016, a retrospective single-centre study compared 915 primary MRI screenings of female breast cancer survivors without additional risk factors to MRI screenings of 606 non-affected women with high genetic or familial risk [45]. The study concluded that specificity was significantly higher (94.0 vs 86.0%, *p* < 0.001) in the survivor compared to the genetic/familial risk group. Furthermore, the false-positive rate (12.3 vs 21.6%, *p* < 0.001) was significantly lower in the survivor subgroup. Sensitivity (80.0 vs 78.6%, *p* > 0.99) and cancer yield (1.7 vs 1.8%, *p* > 0.99) did not differ statistically [45].

MRI surveillance has been suggested to be more beneficial for young breast cancer survivors, particularly those younger than 50 years of age at diagnosis [10, 46]. Women with other recurrence risk factors, such as dense breasts or a first-degree family history, might also benefit more from MRI screening [47]. In its most recent update, the ACR therefore recommended routine annual contrast-enhanced breast MRI surveillance for two subgroups of women with a personal history of breast cancer: women with dense breasts and women with a breast cancer diagnosis before the age of 50. According to the authors, this combination of risk factors is likely to exceed a life-time risk of 20%, justifying annual MRI surveillance as indicated by high-risk screening guidelines [10]. Risk stratification according to patient, treatment and tumour characteristics, will likely transform the selection of imaging modalities and intensity in the near future [48].

### Study limitations

The setup of our study has several limitations worth noting. First of all, the article search and data extraction was performed only by the first author, so lack of double reading could lead to the exclusion of guidelines or data. Secondly, we included only articles with an English-language abstract, although we did not exclude non-English-language articles. Thirdly, we did not use a methodological quality assessment tool to assess the quality of the included practice guidelines.

## Conclusions

Annual surveillance mammography is considered standard practice among guidelines, based on retrospective findings of reduced mortality. Imaging surveillance should not commence earlier than 12 months after diagnosis or 6 months after completion of radiation therapy. No consensus was found regarding intermediate frequency alteration or termination of surveillance.

Although performance of DBT in a surveillance setting is still unclear, the ACR is the first publishing body to recognise DBT as an alternative for breast mammography surveillance. As new evidence emerges, guidelines are likely to undergo revision.

Routine ultrasound surveillance is not recommended by most guidelines. However, optional surveillance is recognised for some subgroups, such as young women or women with dense breasts. Routine breast MRI surveillance is also not recommended, unless women carry additional risk factors, indicating a lifetime recurrence risk > 20%.

## References

[1] Ferlay J, Steliarova-Foucher E, Lortet-Tieulent J (2013). Cancer incidence and mortality patterns in Europe: estimates for 40 countries in 2012. Eur J Cancer.

[2] Belgian Cancer Registry (2016) Incidence fact sheet female breast cancer ICD10: C50 Belgium 2014. Stichting Kankerregister, Brussels. Available via http://www.kankerregister.org/media/docs/Incidencefactsheets/Incidence_Fact_Sheet_FemaleBreastCancer_2014.pdf. Accessed 2 February 2018

[3] Miller KD, Siegel RL, Lin CC (2016). Cancer treatment and survivorship statistics, 2016. CA Cancer J Clin.

[4] Bucchi L, Belli P, Benelli E (2016). Recommendations for breast imaging follow-up of women with a previous history of breast cancer: position paper from the Italian Group for Mammography Screening (GISMa) and the Italian College of Breast Radiologists (ICBR) by SIRM. Radiol Med.

[5] Saslow D, Boetes C, Burke W (2007). American Cancer Society guidelines for breast screening with MRI as an adjunct to mammography. CA Cancer J Clin.

[6] Mainiero MB, Moy L, Baron P (2017). ACR appropriateness criteria® breast cancer screening. J Am Coll Radiol.

[7] National Institute for Health and Care Excellence (NICE) (2017) NICE clinical guideline 164 [CG164]: Familial breast cancer: classification, care and managing breast cancer and related risks in people with a family history of breast cancer. National Institute for Health and Care Excellence (NICE), Manchester. Available via https://www.nice.org.uk/guidance/cg164. Accessed 22 July 2018

[8] Moher D, Liberati A, Tetzlaff J, Altman DG (2009). Preferred reporting items for systematic reviews and meta-analyses: the PRISMA statement. Ann Intern Med.

[9] Moy L, Bailey L, D’Orsi C (2017). ACR appropriateness criteria® stage I breast cancer: initial workup and surveillance for local recurrence and distant metastases in asymptomatic women. J Am Coll Radiol.

[10] Monticciolo DL, Newell MS, Moy L, Niell B, Monsees B, Sickles EA (2018). Breast cancer screening in women at higher-than-average risk: recommendations from the ACR. J Am Coll Radiol.

[11] Runowicz CD, Leach CR, Henry NL (2016). American Cancer Society/American Society of Clinical Oncology breast cancer survivorship care guideline. CA Cancer J Clin.

[12] Khatcheressian JL, Hurley P, Bantug E et al (2013) Breast cancer follow-up and management after primary treatment: American Society of Clinical Oncology clinical practice guideline update. J Clin Oncol 31:961–965 10.1200/JCO.2012.45.9859 23129741

[13] Alberta Provincial Breast Tumour Team (2013) Follow-up care for early-stage breast cancer. Alberta Health Services, Edmonton. Available via https://www.albertahealthservices.ca/assets/info/hp/cancer/if-hp-cancer-guide-br013-early-stage-follow-up.pdf. Accessed 8 July 2018

[14] Guidelines and Protocols Advisory Committee British Columbia Ministry of Health (2013) Breast cancer: management and follow-up. Guidelines and protocols advisory committee British Columbia Ministry of Health, Victoria. Available via http://www2.gov.bc.ca/assets/gov/health/practitioner-pro/bc-guidelines/brcancer.pdf. Accessed 8 July 2018

[15] Canadian Association of Radiologists (2012) Diagnostic imaging referral guidelines: section M: breast disease. Canadian Association of Radiologists. Available via https://car.ca/wp-content/uploads/Breast-disease.pdf. Accessed 8 July 2018

[16] CancerCareManitoba (2017) Surveillance Recommendations and Checklist 2016: Breast Cancer. CancerCareManitoba, Winnipeg. Available via https://www.cancercare.mb.ca/export/sites/default/For-Health-Professionals/.galleries/files/follow-up-care-files/breast/Breast_FollowUp_Guidelines-Not_Receiving_Hormone_Therapy_Jan_2017.pdf. Accessed 8 July 2018

[17] Onkologie L (2017) Leitlinienreport zur S3-Leitlinie Früherkennung, Diagnostik, Therapie und Nachsorge des Mammakarzinoms Version 4.0. Leitlinienprogramm Onkologie, Berlin. Available via https://www.leitlinienprogramm-onkologie.de/fileadmin/user_upload/LL_Mammakarzinom_Langversion_4.0.pdf. Accessed 8 July 2018

[18] Senkus E, Kyriakides S, Ohno S (2015). Primary breast cancer: ESMO clinical practice guidelines for diagnosis, treatment and follow-up. Ann Oncol.

[19] Haute Autorité de Santé (2010) Guide ALD 30: Cancer du sein. Haute Autorité de Santé, Saint-Denis. Available via http://www.has-sante.fr/portail/upload/docs/application/pdf/2010-02/ald_30_gm_ksein_vd.pdf. Accessed 8 July 2018

[20] Haute Autorité de Santé (2015) Dépistage et prévention du cancer du sein. Haute Autorité de Santé, Saint-Denis. Available via http://www.has-sante.fr/portail/upload/docs/application/pdf/2015-04/refces_k_du_sein_vf.pdf. Accessed 8 July 2018

[21] Wildiers H, Stordeur S, Vlayen J et al (2013) Breast cancer in women: diagnosis, treatment and follow-up. Belgian Health Care Knowledge Centre (KCE), Brussels. Available via https://kce.fgov.be/sites/default/files/atoms/files/KCE_143_Breast_cancer_0.pdf. Accessed 8 July 2018

[22] Nationaal Borstkanker Overleg Nederland (2012) The National Breast Cancer Guideline. Knowledge Institute of Medical Specialists, Utrecht. Available via https://richtlijnendatabase.nl/en/richtlijn/breast_cancer/aftercare_and_follow-up.html. Accessed 8 July 2018

[23] National Breast and Ovarian Cancer Centre (2010) Recommendations for follow-up of women with early breast cancer: a clinical practice guideline. National Breast and Ovarian Cancer Centre, Surry Hills. Available via https://canceraustralia.gov.au/publications-and-resources/cancer-australia-publications/recommendations-follow-women-early-breast-cancer-0. Accessed 8 July 2018

[24] Gradishar WJ, Anderson BO, Aft R et al (2018) National Comprehensive Cancer Network (NCCN) Guidelines Version 1.2018 Breast Cancer. National Comprehensive Cancer Network, Fort Washington. Available via https://www.nccn.org/professionals/physician_gls/pdf/breast.pdf. Accessed 8 July 2018

[25] National Institute for Health and Care Excellence (NICE) (2018) NICE guideline 101 [NG101]: Early and locally advanced breast cancer: diagnosis and management. National Institute for Health and Care Excellence (NICE), Manchester. Available via https://www.nice.org.uk/guidance/ng101. Accessed 22 July 2018

[26] New Zealand Guidelines Group (2009) Management of early breast cancer. New Zealand Guidelines Group, Wellington. Available via https://www.health.govt.nz/system/files/documents/publications/mgmt-of-early-breast-cancer-aug09.pdf. Accessed 8 July 2018

[27] The Royal College of Radiologists (2013) Guidance on screening and symptomatic breast imaging, 3rd edn. The Royal College of Radiologists, London. Available via https://www.rcr.ac.uk/system/files/publication/field_publication_files/BFCR%2813%295_breast.pdf. Accessed 8 July 2018

[28] Krishnamurthy R, Whitman GJ, Stelling CB, Kushwaha AC (1999). Mammographic findings after breast conservation therapy. Radiographics.

[29] Lin K, Eradat J, Mehta NH (2008). Is a short-interval postradiation mammogram necessary after conservative surgery and radiation in breast cancer?. Int J Radiat Oncol Biol Phys.

[30] Hymas RV, Gaffney DK, Parkinson BT, Belnap TW, Sause WT (2012). Is short-interval mammography necessary after breast conservation surgery and radiation treatment in breast cancer patients?. Int J Radiat Oncol Biol Phys.

[31] Robertson C, Arcot Ragupathy SK, Boachie C (2011). The clinical effectiveness and cost-effectiveness of different surveillance mammography regimens after the treatment for primary breast cancer: systematic reviews registry database analyses and economic evaluation. Health Technol Assess.

[32] Dunn JA, Donnelly PK, Marshall A (2014). Follow-up in early breast cancer—a surgical and radiological perceptive. Clin Oncol (R Coll Radiol).

[33] Arasu VA, Joe BN, Lvoff NM (2012). Benefit of semiannual ipsilateral mammographic surveillance following breast conservation therapy. Radiology.

[34] de Bock GH, Bonnema J, Zwaan RE, van de Velde CJ, Kievit J, Stiggelbout AM (2004). Patient’s needs and preferences in routine follow-up after treatment for breast cancer. Br J Cancer.

[35] Montgomery DA, Krupa K, Jack WJ (2007). Changing pattern of the detection of locoregional relapse in breast cancer: the Edinburgh experience. Br J Cancer.

[36] Houssami N, Ciatto S (2010). Mammographic surveillance in women with a personal history of breast cancer: how accurate? How effective?. Breast.

[37] Houssami N, Ciatto S, Martinelli F, Bonardi R, Duffy SW (2009) Early detection of second breast cancers improves prognosis in breast cancer survivors. Ann Oncoll 20:1505–1510 10.1093/annonc/mdp037 19297316

[38] Freyvogel M, Padia S, Larson K (2014). Screening mammography following autologous breast reconstruction: an unnecessary effort. Ann Surg Oncol.

[39] Lee JM, Georgian-Smith D, Gazelle GS (2008). Detecting nonpalpable recurrent breast cancer: the role of routine mammographic screening of transverse rectus abdominis myocutaneous flap reconstructions. Radiology.

[40] Lam DL, Houssami N, Lee JM (2017). Imaging surveillance after primary breast cancer treatment. AJR Am J Roentgenol.

[41] Sia J, Moodie K, Bressel M (2016). A prospective study comparing digital breast tomosynthesis with digital mammography in surveillance after breast cancer treatment. Eur J Cancer.

[42] Berg WA, Zhang Z, Lehrer D (2012). Detection of breast cancer with addition of annual screening ultrasound or a single screening MRI to mammography in women with elevated breast cancer risk. JAMA.

[43] Sardanelli F, Boetes C, Borisch B et al (2010) Magnetic resonance imaging of the breast: recommendations from the EUSOMA working group. Eur J Cancer 46:1296–1316 10.1016/j.ejca.2010.02.015 20304629

[44] Weinstock C, Campassi C, Goloubeva O (2015). Breast magnetic resonance imaging (MRI) surveillance in breast cancer survivors. Springerplus.

[45] Lehman CD, Lee JM, DeMartini WB (2016). Screening MRI in women with a personal history of breast cancer. J Natl Cancer Inst.

[46] Cho N, Han W, Han BK (2017). Breast cancer screening with mammography plus ultrasonography or magnetic resonance imaging in women 50 years or younger at diagnosis and treated with breast conservation therapy. JAMA Oncol.

[47] Houssami N, Abraham LA, Kerlikowske K et al (2013) Risk factors for second screen-detected or interval breast cancers in women with a personal history of breast cancer participating in mammography screening. Cancer Epidemiol Biomarkers Prev 22:946–961 10.1158/1055-9965.EPI-12-1208-T PMC3650092 23513042

[48] Witteveen A, Vliegen IM, Sonke GS, Klaase JM, IJzerman MJ, Siesling S (2015). Personalisation of breast cancer follow-up: a time-dependent prognostic nomogram for the estimation of annual risk of locoregional recurrence in early breast cancer patients. Breast Cancer Res Treat.

